# Month of Birth and Mortality in Sweden: A Nation-Wide Population-Based Cohort Study

**DOI:** 10.1371/journal.pone.0056425

**Published:** 2013-02-15

**Authors:** Peter Ueda, Anna-Karin Edstedt Bonamy, Fredrik Granath, Sven Cnattingius

**Affiliations:** Clinical Epidemiology Unit, Department of Medicine, Solna, Karolinska Institutet, Stockholm, Sweden; University of Turku, Finland

## Abstract

**Background:**

Month of birth – an indicator for a variety of prenatal and early postnatal exposures – has been associated with life expectancy in adulthood. On the northern hemisphere, people born in the autumn live longer than those born during the spring. Only one study has followed a population longitudinally and no study has investigated the relation between month of birth and mortality risk below 50 years.

**Methods and results:**

In this nation-wide Swedish study, we included 6,194,745 subjects, using data from population-based health and administrative registries. The relation between month of birth (January – December) and mortality risk was assessed by fitting Cox proportional hazard regression models using attained age as the underlying time scale. Analyses were made for ages >30, >30 to 50, >50 to 80 and >80 years. Month of birth was a significant predictor of mortality in the age-spans >30, >50 to 80, and >80 years. In models adjusted for gender and education for ages >30 and >50 to 80 years, the lowest mortality was seen for people born in November and the highest mortality in those born in the spring/summer, peaking in May for mortality >30 years (25‰ excess hazard ratio compared to November, [95% confidence interval = 16–34 ]) and in April for mortality >50 to 80 years (42‰ excess hazard ratio compared to November, [95% confidence interval = 30–55]). In the ages >80 years the pattern was similar but the differences in mortality between birth months were smaller. For mortality within the age-span >30 to 50 years, results were inconclusive.

**Conclusion:**

Month of birth is associated to risk of mortality in ages above 50 years in Sweden. Further studies should aim at clarifying the mechanisms behind this association.

## Introduction

A large body of epidemiological evidence suggests that risks of diseases and mortality in adult age is not only determined by genetics and lifestyle but also by factors acting in early life.[1]–[3].

Month of birth is frequently used as a proxy for a wide array of prenatal and early postnatal environmental exposures related to season. These include nutritional status, ambient temperature, sun exposure, exposure to infections and other environmental influences. [4], [5] Observations from the northern hemisphere demonstrate that people born in the autumn (October-December) live longer than those born during spring (April-June).[6]–[8].

Whether the effect of month of birth on mortality risk change during the life course is of interest to clarify, as risks of diseases and prevailing causes of deaths vary between age-spans. Most studies have only indirectly studied the influence of month of birth on mortality by cross-sectional reviews of death certificates from which average age at death has been calculated. [7], [9] To our knowledge, there is only one longitudinal population-based study that has investigated the association between month of birth and mortality. In a closed cohort, more than 1.3 million Danes aged 50 years or more were followed for 30 years. Paralleling the findings of the cross-sectional studies, subjects born in autumn were shown to have a longer remaining life expectancy compared to spring born subjects. [8].

In this nation-wide study, we included over 6 million subjects who lived in Sweden in 1991 and who were more than 30 years sometime during the follow-up time of 20 years. We assessed the relation between month of birth and mortality in the ages >30, >30 to 50, >50 to 80 and >80 years.

## Methods

### Study Population and Data Sources

Data used in the study was obtained from population-based health and administrative registries in Sweden. Cross-linkage across these registries was possible using the person-unique Personal Identification Number (PIN), assigned to all Swedish residents [10]. Using the Swedish Total Population Register, we identified all Swedish-born subjects who were living in the country on the 1^st^ of January 1991. We included all subjects who were more than 30 years old before the end of follow up (December 31^st^, 2010; n = 6,583,693). From the Swedish Total Population Register, we also retrieved information about dates of emigration and death. Data on subjects’ educational level - categorized into primary, secondary and higher education (studies at university) - was obtained from The Education Register for the years 1990, 1995, 2000, 2005 and 2010. The highest educational level reached during the study time was defined as the subject’s education. Subjects were followed until death, emigration or end of follow-up (December 31^st^ 2010). The study was approved by the regional ethics committee in Stockholm (Dnr 19803/2011). The committee waived the need for written consent from the participants.

### Statistical Methods

The population was divided by month of birth into twelve groups. To adjust for cohort effects, all analyses were stratified on 10-year birth cohorts. The relation between quarter of birth (January-Mars, April-June, July-September, and October-December), and education status was assessed with Chi-2 test. Quarter of birth year was used in order to decrease the number of comparisons in the test.

We assessed the association between month of birth and mortality by fitting Cox proportional hazards regression models using attained age as the underlying time scale. The analyses only included ages above 30 years. Left truncation was adjusted for by calculating an individual’s age on the 1^st^ of January 1991. Subjects contributed with person-time only in the age-spans that they belonged to during the study time. For example, a subject who was 20 years at the start of the study entered the study at the age of 30, i.e. 10 years after start of study follow-up, and contributed with person-time during the 10 years of remaining study time, between the age of 30 and 40.

As previous studies have shown lowest mortality for subjects born in November, [8] this month was used as reference in the Cox regression analyses. Relative risks are presented as the excess hazard ratio (EHR) in per mille ((Hazard ratio –1)*1000). Analyses were first conducted for the ages of >30 years and separate analyses were then performed for the age-spans >30 to 50, >50 to 80 and >80 years. The crude models were followed by analyses adjusted for sex and education. Due to the computational burden, it was not possible to statistically test the differences in month of birth effects on mortality between the chosen age-spans. For ages >30 years, sex stratified analyses adjusted for education were also performed.

Six percent (n = 388,948) of the subjects lacked education data. We therefore compared results from crude analyses of month of birth and mortality in the age-spans >30, >30 to 50, >50 to 80 and >80 years, when including and excluding subjects with missing information on education. As the influence of month of birth on mortality risks did not essentially differ between the models, subjects without education data were excluded in the analyses presented in the study (Figure S1 provides information on mortality >30 years in all subjects as compared to subjects with data on education, results from other comparisons are available on request).

In post-hoc analyses, we also investigated potential interaction effects between month of birth and education, as well as month of birth and sex in the age-span >30 years. Interaction variables were created for education/sex categories (three and two categories, respectively) and the four months with the highest and lowest hazard ratios respectively. In complementary analyses, we also tested for differences in mortality between subjects born in December and January. Statistical analyses were conducted in SAS version 9.0 (SAS Institute, Cary, North Carolina, USA).

## Results

A total of 6,194,745 subjects contributed with person-time to the study. During the time of follow-up, 1,287,927 (20.8%) of the subjects died. The proportion of the population that died during the study time increased with age-span, male sex and lower education. (Table 1).

**Table 1:** Number of subjects and proportion of the population that died during the study time by age-span, sex and education group.

	>30		>30 to 50		>50 to 80		>80	
	Subjects (n = 6,194,745)	Deaths (n = 1,287,927)	Subjects (n = 3,989,786)	Deaths (n = 47,595)	Subjects (n = 4,240,338)	Deaths (n = 655,532)	Subjects (n = 1,026,435)	Deaths (n = 584,611)
*Sex*		Proportion dead (%)		Proportion dead (%)		Proportion dead (%)		Proportion dead (%)
Male	3,095,006	22.0	2,047,535	1.5	2,080,300	18.7	426,597	61.3
Female	3,099,739	19.6	1,942,251	0.9	2,160,038	12.3	599,838	53.9
*Education*								
Primary	1,975,389	41.5	669,714	2.2	1,788,988	21.8	676,780	61.4
Secondary	2,566,376	13.7	1,926,097	1.3	1,590,157	12.5	258,865	50.1
Higher	1,652,980	7.0	1,393,975	0.6	861,193	7.8	90,790	43.3

Quarter of birth year was significantly associated to education status (p<0.001, Chi-Square = 1559.06, degrees of freedom = 6). Higher education was slightly more common among subjects born from January to June (monthly rates ranged from 27 to 28%) than among subjects born from July to December (25–26%). Subjects born from January to June were consequently less likely to have only secondary education (41% in all months) and primary education (31–32%) than subjects born from July to December (secondary education: 42% in all months; primary education: 32–33%).

In the total population above 30 years of age, month of birth predicted mortality in the crude model, and adjusting for sex and education did not attenuate these associations. Compared to November, being born in January to September was associated with significantly increased mortality, and the excess hazard ratio was highest from April through July. When the analyses were stratified by sex (and adjusted for education), the patterns were similar, although there were slight sex differences with respect to month of birth and mortality risk. The lowest mortality risks were seen in October or November in all analyses (Table 2).

**Table 2:** Number of deaths and excess hazard ratio (EHR) in ‰ compared to November (95% confidence interval) in the age-span >30: crude model, adjusted for sex and education, male (adjusted for education) and female (adjusted for education) and with p-value for type 3-test.

	Number of deaths (n = 1,287,927)	Crude		Adjusted for sex and education		Male		Female	
		EHR ‰	95% CI	EHR ‰	95% CI	EHR ‰	95% CI	EHR ‰	95% CI
January	109,186	13	4 to 22	14	5 to 23	14	2 to 26	14	1 to 27
February	103,590	11	2 to 20	14	5 to 23	16	4 to 28	12	−1 to 25
Mars	118,627	12	3 to 20	14	5 to 23	14	2 to 26	14	2 to 27
April	113,407	19	11 to 28	23	14 to 32	25	13 to 38	20	7 to 33
May	114,741	20	11 to 28	25	16 to 34	26	14 to 38	23	11 to 36
June	106,019	16	7 to 25	21	12 to 30	27	15 to 40	13	1 to 26
July	106,594	21	12 to 30	23	14 to 32	19	7 to 32	27	14 to 40
August	104,524	17	8 to 26	19	10 to 28	20	8 to 32	18	5 to 31
September	109,333	10	1 to 19	11	2 to 20	12	0 to 24	10	−3 to 23
October	102,188	2	−7 to 11	3	−6 to 12	6	−6 to 19	−1	−14 to 12
November	96,229	0 (ref)	ref	0 (ref)	ref	0 (ref)	Ref	0 (ref)	ref
December	103,489	5	−4 to 14	6	−3 to 14	5	−7 to 18	6	−7 to 19
P		<0.001		<0.001		<0.001		<0.001	
Degrees of freedom		11		11		11		11	
Chi –Square (Likelihood Ratio)		53.4873		75.6558		44.7231		38.4738	
Chi –Square (Score)		53.4316		75.5744		44.6961		38.452	
Chi –Square (Wald)		53.2639		75.329		44.6145		38.3862	

We found no interaction effects between sex and month of birth and mortality risk in a model where interaction variables were created with the four months of highest (April–July) and lowest (September–December) mortality risks in the crude analysis (p = 0.518 Chi-square = 1.3171, degrees of freedom = 2). In a similar model, there was a significant interaction between education and month of birth and mortality (p = 0.035 Chi-Square = 10.3668, degrees of freedom = 4). The interaction was derived mainly from a lower month of birth-effect on mortality in the high risk months for the middle education group compared to the low and high education group (data not shown).

**Figure 1:**
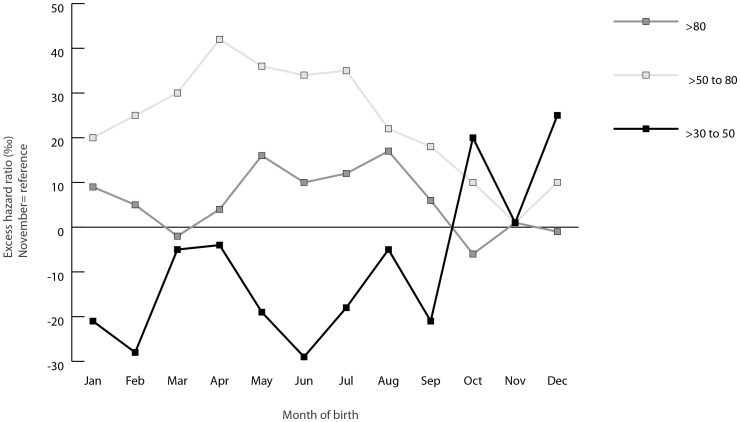
Excess hazard ratio by month of birth. Models for age-spans >30 to 50, >50 to 80 and >80 years presented as ‰ compared to November and adjusted for education and sex.

Associations between month of birth and age-specific mortality are illustrated in Table 3 and Figure 1. Month of birth was not a significant predictor of mortality in the age-span >30 to 50 years, but predicted mortality both at >50 to 80 and >80 years (Table 3). Peak mortality was seen for birth month April in ages >50 to 80 years and for August in ages >80 years. Overall, the mortality was highest for subjects born during the spring and summer months in both age-spans and lowest for subjects born in October-December. (Table 3 and Figure 1). There were no significant differences in mortality risks between December and January in any of the investigated age-spans (data not shown).

**Table 3:** Number of deaths and excess hazard ratio (EHR) in ‰ compared to November (95% confidence interval) in the age-spans >30 to 50, >50 to 80 and >80 years: crude models and adjusted for sex and education with p-value for type 3-test.

	>30 to 50					>50 to 80					>80				
	Number of deaths (n = 47,595)	Crude		Adjusted		Number of deaths (n = 655,532)	Crude		Adjusted		Number of deaths (n = 584,611)	Crude		Adjusted	
		EHR ‰	95%CI	EHR ‰	95%CI		EHR ‰	95%CI	EHR ‰	95%CI		EHR ‰	95%CI	EHR ‰	95%CI
January	3,842	−17	−61 to 29	−21	−64 to 25	53,849	18	6 to 31	20	8 to 33	51,477	9	−4 to 22	9	−4 to 22
February	3,654	−33	−76 to 13	−28	−72 to 18	51,538	21	8 to 34	25	13 to 38	48,381	4	−9 to 17	5	−8 to 18
Mars	4,472	−13	−55 to 32	−5	−47 to 40	60,173	27	15 to 39	30	18 to 42	53,963	−3	−15 to 10	−2	−15 to 11
April	4,449	−14	−57 to 30	−4	−47 to 41	58,414	38	25 to 50	42	30 to 55	50,524	2	−11 to 15	4	−9 to 17
May	4,299	−30	−72 to 14	−19	−62 to 26	59,210	30	18 to 43	36	24 to 49	51,218	12	−1 to 25	16	3 to 29
June	3,910	−32	−75 to 13	−29	−72 to 16	55,138	30	17 to 42	34	21 to 46	46,957	5	−8 to 18	10	−3 to 24
July	3,950	−17	−60 to 29	−18	−61 to 28	55,444	33	21 to 46	35	23 to 48	47,189	10	−3 to 23	12	−1 to 25
August	3,843	−3	−47 to 43	−5	−49 to 42	53,567	21	8 to 33	22	10 to 35	47,098	15	1 to 28	17	4 to 31
September	3,867	−21	−65 to 25	−21	−64 to 25	55,449	18	5 to 30	18	6 to 30	50,004	5	−8 to 17	6	−7 to 19
October	3,893	21	−24 to 69	20	−26 to 67	51,677	9	−3 to 22	10	−3 to 22	46,605	−7	−20 to 6	−6	−19 to 7
November	3,575	0 (ref)	ref	0 (ref)	ref	48,544	0 (ref)	Ref	0 (ref)	ref	44,095	0 (ref)	ref	0 (ref)	ref
December	3,841	30	−16 to 78	25	−20 to 73	52,529	9	−4 to 21	10	−2 to 23	47,100	−2	−15 to 11	−1	−14 to 12
P		0.115		0.287			<0.001		<0.001			0.029		0.003	
Degrees of freedom		11		11			11		11			11		11	
Chi –Square (Likelihood Ratio)		16.3981		12.8955			71.0564		91.2901			21.4824		28.0583	
Chi –Square (Score)		16.4906		12.9473			70.9598		91.1583			21.4823		28.0622	
Chi –Square (Wald)		16.768		13.1022			70.6689		90.7603			21.4818		28.0738	

## Discussion

In this large population-based study, we found that month of birth was a significant predictor of mortality in the age-spans >30, >50 to 80 and >80 years. The highest mortality was seen for people born in the spring/summer, peaking in April (>50 to 80 years, adjusted model), May (>30 years) or August (>80 years), with a corresponding trough in the autumn. In the age-span >30 to 50 years, results were inconclusive.

As for the ages above 50 years, the findings of this study are in broad agreement with previous reports from the northern hemisphere: autumn-born have a survival advantage compared to those born in the spring and summer. Seemingly, these effects were more pronounced in the age-span >50 to 80 years compared to >80 years. One possible explanation for this is selective mortality: irrespective of month of birth, the frailer will die first. With increasing age, the remaining population will constitute an increasingly homogenous group of robust individuals, and differences in mortality risk by month of birth will therefore gradually diminish. [9].

Possible explanations for the association between month of birth and mortality after 50 years have been scrutinized by others. [9] Seasonal differences in nutrition availability and infections have been proposed as the main causes of the observed correlations. [9] In the beginning of the 20^th^ century, food supply, especially fruit and vegetables, was much dependent on season. Mothers to children born in the autumn are likely to have had a better nutritional status during pregnancy - especially during the third trimester when the peak growth in fetal weight occurs [11] – as compared to the mothers of spring-born children. Evidence linking nutrition in utero to adult disease is supportive of this hypothesis. [2] Seasonality in birth weight – an indicator of fetal nutrition and a controversial risk factor for adult disease – has been observed in different parts of the world [12] and attributed to both nutrition [13] and the load of infectious diseases on the mother during the third trimester of pregnancy. [14], [15] Several ecological studies have observed that cohorts exposed to infections in early life are at increased risk for disease and death in late life. [16], [17].

Recently, vitamin D-deficiency has been highlighted as an early life nutritional factor of potential importance to health later in life. It has been suggested that the prenatal period constitutes a critical window during which vitamin D-deficiency may predispose the fetus for adult-onset disease. [18] Although there are small contributions from diet and supplements, skin exposure to ultraviolet light (UVB) is the main determinant for vitamin D-levels in humans. [19] On high latitudes, vitamin D levels are higher in both mothers and neonates during the summer as compared to the winter. [20], [21] It can thus be hypothesized that individuals born in the spring in Sweden – a country with large differences in sunlight hours between the seasons- are exposed to lower levels of vitamin D in fetal life, in particular during the last two trimesters, compared to those born in the autumn.

In studies on rodents, fetal vitamin D-deficiency has led to retarded cardiac development [22] and alterations in the kidney development, possibly translated to elevated blood pressure [23]. Maternal vitamin D-deficiency in the third trimester in an Indian population was linked to higher fasting insulin resistance and smaller arm muscle area at the age of 9.5 years in the offspring. [24] In England, low vitamin D during pregnancy in mothers predicted adiposity in their 6 year old children. [25] Low vitamin D during gestation has also been associated to risks of schizophrenia [26] and multiple sclerosis [27]. Given the high prevalence of vitamin D-deficiency in pregnant women as seen today in many parts of the world [21], [28], [29], its potential role in adult-age mortality is of interest to investigate further.

Socioeconomic status - in this study indicated by educational level - is of importance to consider in month of birth studies. Socioeconomic status is a life course factor affecting disease and mortality risks. Preferences regarding the timing of having children may vary between couples of different socioeconomic groups. Month of birth-patterns in mortality may simply reflect such socioeconomic differences in seasonality of birth [30], [31], and socioeconomic factors are therefore potential confounders. In our study, those born in the first half of the year were more likely to have a higher education than subjects born in the second half of the year. However, the association between month of birth and mortality remained after controlling for education. Moreover, educational level of an individual is closely linked to the educational level of the parents. [32], [33] As parents in low socioeconomic groups may have been more affected by seasonal fluctuations in nutrition availability in the beginning of the 20^th^ century than parents in high socioeconomic groups, education can also be seen as an indicator for the perinatal environment. The effects of month of birth on adult mortality would in this case interact with education. However, the interaction effect found in our study was in the form of a lower month of birth-effect on mortality risk for the group with secondary education compared to the low and high education group. This is not in line with any known hypothesis and could be a chance finding.

Cross-sectional studies have found that an effect of month of birth is present for a diverse range of causes of death, including cardiovascular disease and malignant neoplasms [9], which are the major causes of death after 50 years in Sweden [34]. It is therefore feasible to assume that these month of birth effects are also present in our study population. In the youngest age-span of this study (>30 to 50 years), suicide, accidents and other causes of death related to social factors are more prominent [35]. Apart from the relatively low number of deaths occurring in this age-span compared to older ages, different pathways between month of birth and mortality could explain the inconclusive and different month of birth-pattern seen in ages >30 to 50 years as compared to >50 to 80 and >80 years.

This study, comprising observations from over 6 million subjects, is the largest longitudinal population-based study assessing the relation between month of birth and mortality in adult age. It is also the first study in the field including longitudinal data from age-spans below 50 years. By excluding people born outside Sweden, we were able to create a homogenous study population with respect to seasonally dependent early life exposures as well as country-specific social factors. Further, we had access to information about subjects’ education (a potential confounder) in our analyses.

Generalizing the findings to other parts of the world may not be strictly possible, given the study setting being confined to Sweden, the Swedish climate and its specific seasonal exposures. Our findings regarding the older age-spans, however, are in line with other reports from the northern hemisphere.

Due to the large number of subjects included in this study, we were able to detect very small risk differences between subjects born in different months that are hard to translate into biological or clinical relevant measures. The relevance of this study could thus be considered as providing clues for the identification of early life exposures of potential public health interest.

In conclusion, this longitudinal population-based study assessed the association between month of birth and mortality in different age-spans in more than 6 million Swedes. Confirming previous findings from the northern hemisphere, spring/summer-born were shown to die sooner than autumn-born in ages 50 years and above. The associations remained after controlling for sex and education. Future studies on the topic should focus on elucidating the mechanisms underlying these observations.

## Supporting Information

Figure S1 **Excess hazard ratio by month of birth.** Including all Swedish-born subjects living in the country on the 1st of January 1991 and excluding subjects without education data (‰ compared to November).
(TIF)
