# Supplementary figures and images for: Month of Birth and Mortality in Sweden: A Nation-Wide Population-Based Cohort Study
Source: PLoS One. 2013 Feb 15;8(2):e56425. doi: 10.1371/journal.pone.0056425 (PMC3574007; doi:10.1371/journal.pone.0056425)

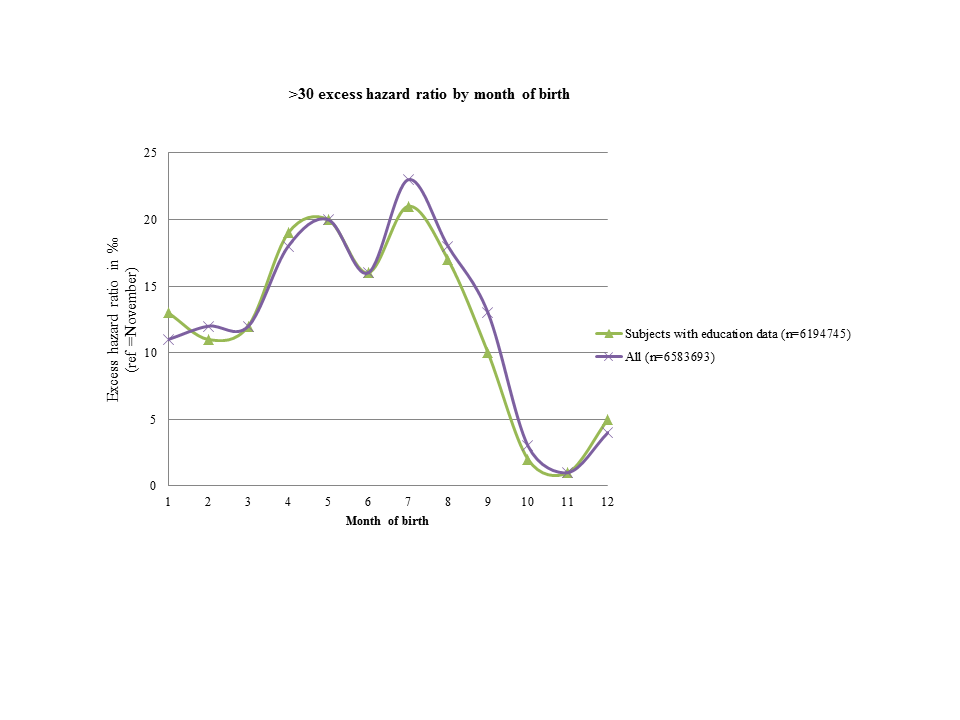

Supplement: Figure S1 — Excess hazard ratio by month of birth. Including all Swedish-born subjects living in the country on the 1st of January 1991 and excluding subjects without education data (‰ compared to November). (TIF) [file pone.0056425.s001.tif]
